# Cost-effectiveness analysis of quadrivalent influenza vaccine versus trivalent influenza vaccine for elderly in Hong Kong

**DOI:** 10.1186/s12879-014-0618-9

**Published:** 2014-11-25

**Authors:** Joyce HS You, Wai-kit Ming, Paul KS Chan

**Affiliations:** School of Pharmacy, Faculty of Medicine, The Chinese University of Hong Kong, Shatin, NT Hong Kong; Department of Microbiology, Faculty of Medicine, The Chinese University of Hong Kong, Shatin, NT Hong Kong; Stanley Ho Centre for Emerging Infectious Diseases, Faculty of Medicine, The Chinese University of Hong Kong, Shatin, NT Hong Kong

**Keywords:** Quadrivalent influenza vaccine, Trivalent influenza vaccine, Elderly, Hong Kong, Cost-effectiveness analysis

## Abstract

**Background:**

Cost and quality-adjusted life-years (QALYs) gained by quadrivalent influenza vaccine (QIV) versus trivalent influenza vaccine (TIV) in Hong Kong elderly were estimated over 9 seasons.

**Methods:**

TIV-unmatched influenza B infection rates with QIV versus TIV were estimated by an epidemiology model. Model parameters included percentages of influenza B lineages in circulation, influenza B-associated hospital admission, age-specific population, vaccine coverage and effectiveness. Incremental cost per QALY gained (ICER) by QIV versus TIV were estimated from Hong Kong’s societal perspective.

**Results:**

Mean reduction in influenza B infection rate was 191.3 (95%CI 45.1-337.5) per 100,000 population aged ≥65 years. Highest cost savings and QALYs gained by QIV occurred in 2007 with high percentage of TIV-unmatched strain (92.9%) for age groups 65–79 years (USD266,473 and 22.8 QALYs) and ≥80 years (USD483,461 and 27.3 QALYs). ICERs of QIV were below willingness-to-pay for age group 65–79 years in 6, 5 and 3 years when QIV cost + USD1 + USD2 and + USD5 more than TIV, respectively. For age group ≥80 years, ICERs of QIV were below willingness-to-pay in 7 and 5 years when QIV cost + USD1 and + USD5, correspondingly.

**Conclusions:**

Acceptance of QIV to be cost-effective in Hong Kong elderly was subject to QIV unit cost and percentage of circulating TIV-unmatched influenza B lineages.

## Background

We recently reported the percentages of influenza B lineages (B/Victoria and B/Yamagata) in clinical isolates in 2000–2010 and found that co-circulation of both influenza B lineages was very common in Hong Kong [1]. Furthermore, one of the influenza B lineages predominated (accounting for over 80% of the circulating influenza B strains) in six of the ten years. These predominated years were associated with increased risk of hospitalization by 1.4-fold. The seasonal trivalent influenza vaccine (TIV) covers two subtypes of influenza A (A/H1N1 and A/H3N2) plus one of the two influenza B lineages circulating in humans, and TIV only matched the predominated influenza B strain in two of these six predominated years.

Findings in literature suggested that including two lineages of influenza B in a quadrivalent influenza vaccine (QIV) would have a positive impact in terms of cost saving and reducing hospitalization related to influenza infection [2],[3]. To further quantify the clinical and economic impact of QIV, we estimated the difference in costs and quality-adjusted life-years (QALYs) gained by QIV, when compared to TIV, in elderly population over the period of 2001–2010 from a societal perspective of Hong Kong.

## Methods

In the present analysis, we estimated the potential cost and QALYs difference of influenza B, when compared to TIV, from 2001–2010 years in elderly population (aged ≥65 years) in Hong Kong. Year 2009 was excluded as it was dominated by the novel pandemic influenza A 2009 H1N1. Events caused by infection of the TIV-unmatched influenza B lineage in each year were calculated by an epidemiology model. The infections caused by the influenza B lineage or influenza A subtypes covered by both TIV and QIV were excluded, assuming that there were no change in the infection rates. The costs and QALYs loss for symptomatic patients who received medical care in outpatient and hospital setting were estimated from the event rates. The parameters used for estimation of clinical events, cost difference and QALYs gained by QIV versus TIV were listed in Table 1. All model parameters were obtained from published literature.

**Table 1:** **Parameters for calculation of event rates, costs and QALYs with QIV versus TIV**

Parameter	Values	References
*Clinical inputs*		
Influenza vaccine coverage (≥65 years)	39.1%	15
TIV vaccine effectiveness against influenza B (2001–2010)	0.45-0.75	7-14
Reduction in TIV influenza B vaccine effectiveness against TIV-unmatched influenza B lineage	30%	6
Influenza B hospitalization rate (2001–2010) by age group (per 100,000):		1
65-79 years	1-40.8	
≥ 80 years	0-222.1	
Hong Kong population (2001−2010) by age group:		24
65-79 years	600,986-665,642	
≥ 80 years	146,066-257,966	
Influenza B lineage in circulation (2001−2010)		1
Yamagata	5.3%-96.3%	
Victoria	3.7-94.7%	
Case-fatality ratio	0.05%	5
Percent of inpatient cases with ICU admission:	2.4%	4
Mortality rate of influenza B in ≥65 years inpatient cases	5.9%	1
*Utility*		
Utility score for >65 years	0.84	25
Utility loss		16-22
Outpatient care	0.40	
Hospitalization without ICU care	0.50	
Hospitalization with ICU care	0.62	
Life expectancy in Hong Kong (years)	83	24
Cost (USD)*		
Cost of outpatient clinic (per visit)	49	26
Number of clinic visit for patients without hospitalization	1	Assumption
Cost of hospitalization without ICU care (per day)	600	26
Cost of hospitalization with ICU care (per day)	2,949	26
Length of illness (days)		
Outpatient care	7	23
Hospitalization (length of stay)	10.8	4
Caregiver salary per day	47	24
Labor force rate	68%	24
Unemployment rate	3.7%	24

*1 USD =7.8 HKD.

### Clinical events analysis

For each year, the hospitalization rate of target B lineage was calculated using the influenza B hospital admission rate and the percentage of target influenza B lineage identified in the isolates reported in our previous cohort study [1]. Briefly, in this prior study we identified hospitalized patients with laboratory confirmed influenza virus A or B infection and the circulation pattern of influenza B lineage in the Prince of Wales Hospital (a 1300-bed Hong Kong teaching hospital), and calculated age-stratified population-based hospitalization rates of influenza A and B for years from 2000–2010 (excluding 2009 for the same reason above).

The intensive care unit (ICU) admission rate was calculated by hospitalization rate of target lineage together with ICU admission rate among hospitalized patients [4]. The mortality rate of influenza B among hospitalized patients was retrieved from patients aged ≥65 years in the same cohort of our previous study [1]. The expected annual infection rate of target lineage was estimated using the mortality rate and a case-fatality ratio of influenza [5]. The rate of outpatient care was further calculated as the difference of estimated annual infection rate and hospitalization rate. To estimate the reduction in event rates of outpatient care, hospitalization, ICU admission and death with the use of QIV versus TIV, the epidemiology model described by Reed et al. was adoped [2]:

1. Expected event rate without influenza vaccination = Event rate with TIV/(1 − (VC × VE_TIV_))
2. Expected event rate with QIV = expected event rate without influenza vaccination × (1× (VC × VE_QIV_)) where VC and VE were the vaccine coverage and vaccine effectiveness against the TIV-unmatched lineage, respectively.
3. Reduction in event rate = Event rate with TIV − expected event rate with QIV.

The VE_TIV_ against the TIV-unmatched influenza B lineage was estimated to be reduced by 30% [6]. The VE_QIV_ against the TIV-unmatched influenza B lineage was assumed to be the same as VE of TIV against the TIV-selected influenza B lineage, and the year-specific VE was retrieved from literature reported in the period of 2001–2010 [7]-[14]. The year 2012 age-specific influenza vaccine coverage rate (39.1%) in elderly published by the Department of Health of Hong Kong was applied in the present analysis for 9 seasons [15].

The reduction in case numbers of each event (outpatient care, hospitalization, ICU admission and death) were then calculated by multiplying the change in event rate and the population of ≥65 years for each year.

### QALY analysis

The QALYs gained by QIV were calculated using the number of reduced infected cases, and the utility value and duration of time-spent in each of the four statuses: (1) Outpatient care, (2) hospitalization without ICU admission, (3) ICU care, and (4) death. The utilities were retrieved from health-related quality of life reported from literature (Table 1) [16]-[22]. The time-spent in outpatient care and hospitalization were the length of outpatient duration of illness [23] and length of hospital stay [4], correspondingly. The QALYs loss of death was estimated using life expectancy in Hong Kong and age-specific utility value for ≥65 years [24],[25]. The QALYs loss in both vaccine arms was discounted to 2014 with 3% annual discounted rate.

### Cost analysis

Both direct medical cost and indirect cost of reduced cases of the TIV-unmatched influenza B lineage were considered as cost savings of QIV, from the societal perspective of Hong Kong. Direct medical cost included costs of outpatient care and hospitalization. The cost per outpatient clinic visit and cost per hospital day (with and without ICU care) were estimated from the 2014 charges of the Hospital Authority (HA). HA is the largest, non-profit-making public health organization in Hong Kong. Assuming the charges listed by the Hospital Authority represent only the cost components (including labor costs) with no addition of profits, the cost per general outpatient visit and daily cost of hospitalization were therefore approximated using the Hospital Authority charges [26].

Productivity loss of caregivers of elderly patients was estimated by the median daily income (non-gender specific) of the population in Hong Kong [24], and duration of hospitalization for inpatient care. For patients receiving only outpatient care, the duration of productivity loss was limited to days attending outpatient clinic. The number of clinic visit was assumed to be one time for a conservative estimation on both direct and indirect costs of outpatient care. The proportion of employed caregivers was estimated by the percentage of Hong Kong population in the labor force and the employment rate among the labor force [24].

### Cost-effectiveness analysis

The incremental cost per QALY gained (ICER) by QIV versus TIV was calculated using the following equation:

(Additional vaccine cost of QIV versus TIV – Cost-savings of QIV)/QALYs gained by reduced events, where additional vaccine cost of QIV was calculated by:

Additional unit cost of QIV versus TIV × age-specific coverage rate × population of age group

The current unit cost of TIV in Hong Kong is USD7.7. Four levels of additional cost (the unit cost of QIV to be USD1, 2, 5 and 10 more than TIV) were considered in the cost-effectiveness analysis. As recommended by the World Health Organization (WHO), three-fold of gross domestic product (GDP) per capita was used as the threshold of willingness-to-pay per QALY [27]. A scenario with ICER of QIV less than 3-fold of GDP per capita of Hong Kong was considered as cost-effective.

## Results

The reduction in expected events rates of TIV-unmatched influenza B by QIV versus TIV are shown in Table 2. The mean reduction in influenza infection rate for elderly aged ≥65 years was 191.3 (95%CI 45.1-337.5) per 100,000 in 2001–2010. Age-specific reduction in the age group 65–79 years was 104.8 (95%CI 27.2-182.4) per 100,000, and 451.4 (95%CI 87.7-815.1) per 100,000 in population aged ≥80 years.

**Table 2:** **Reduction in expected event rates of TIV-unmatched influenza B by QIV versus TIV in Hong Kong elderly**

Year	Rate reduction (per 100,000)			
	Symptomatic influenza	Outpatient	Hospitalization	Dead
**Age 65–79 years**				
2001	2.6809	2.6582	0.0227	0.0013
2002*	0.0000	0.0000	0.0000	0.0000
2003	3.7933	3.7611	0.0321	0.0019
2004	282.0652	279.6748	2.3904	0.1410
2005	84.4697	83.7538	0.7158	0.0422
2006	27.5809	27.3472	0.2337	0.0138
2007	282.9799	280.5818	2.3981	0.1415
2008	201.6311	199.9223	1.7087	0.1008
2010	58.1889	57.6958	0.4931	0.0291
**Age ≥80 years**				
2001	1.9449	1.9285	0.0165	0.0010
2002*	0.0000	0.0000	0.0000	0.0000
2003	42.1055	41.7487	0.3568	0.0211
2004	838.6402	831.5331	7.1071	0.4193
2005	204.2730	202.5419	1.7311	0.1021
2006	49.7802	49.3583	0.4219	0.0249
2007	1540.4373	1527.3828	13.0546	0.7702
2008	1032.5602	1023.8097	8.7505	0.5163
2010	352.9492	349.9581	2.9911	0.1765

*In 2002, no influenza B virus was identified from patients admitted to the Prince of Wale Hospital for age groups 65–79 years and ≥80 years. The expected reductions in event rates were considered as zero.

The highest total cost savings and QALYs gained by QIV in age group 65–79 years occurred in year 2007 (USD266,473 and 22.8 QALYs), followed by year 2004 (USD256,127 and 21.9 QALYs), and year 2008 (USD191,105 and 16.4 QALYs) (Table 3). Similarly, QIV had the highest cost-saving and QALYs gained from reduced cases of influenza B in age group ≥80 years in 2007 (USD483,461 and 27.3 QALYs), year 2008 (USD344,103 and 19.5 QALYs) and year 2004 (USD218,829 and 12.4 QALYs). In the year 2002, no influenza B virus was identified in hospital admissions at the Prince of Wales Hospital for age groups 65–79 years and ≥80 years. The expected reductions in infection, cost-saving and additional QALYs gained by QIV were considered to be zero in 2002.

**Table 3:** **QALY gained and cost savings of QIV versus TIV**

Year	QALYs	Direct cost	Indirect cost	Total cost
**Age 65–79 years**				
2001	0.199	1,781	543	2,324
2002*	0	0	0	0
2003	0.291	2,600	792	3,392
2004	21.940	196,311	59,816	256,127
2005	6.670	59,679	18,184	77,863
2006	2.210	19,777	6,026	25,803
2007	22.826	204,241	62,232	266,473
2008	16.370	146,474	44,631	191,105
2010	4.785	42,818	13,047	55,864
**Age ≥80 years**				
2001	0.023	314	96	410
2002*	0	0	0	0
2003	0.581	7,880	2,401	10,281
2004	12.372	167,723	51,105	218,829
2005	3.207	43,477	13,247	56,724
2006	0.829	11,234	3,423	14,657
2007	27.335	370,553	112,907	483,461
2008	19.455	263,741	80,362	344,103
2010	7.425	100,651	30,668	131,319

*In 2002, no influenza B virus was identified from patients admitted to the Prince of Wale Hospital for age groups 65–79 years and ≥80 years. The expected reductions in infected cases, cost-saving and additional QALYs gained with QIV were considered as zero.

The GDP per capita of Hong Kong was USD36,557.^32^ Using 3-time GDP per capita (USD109,671) as the threshold of willingness-to-pay, ICERs of QIV indicated that it was more cost-effective than TIV for age group 65–79 years in 6 years when QIV cost USD1 more than TIV (Table 4). When QIV was more costly than TIV by USD2, USD5 and USD10, it was more cost-effective than TIV in 5, 3 and 2 years, respectively. In the age group ≥80 years, the ICERs of QIV was below the threshold of willingness-to-pay in 7 years when QIV versus TIV cost level was + USD1. QIV remained to be cost-effective in 5 years when it cost up to USD5 more than TIV. It was cost-effective in 3 years when it cost USD10 more than TIV.

**Table 4:** **ICER** ^**#**^ **of QIV versus TIV**

Year	ICER	
Age (years)	65-79	≥80
***Additional cost of QIV = USD$1***		
2001	1,168,835	2,447,575
2002	NE*	NE*
2003	822,641	**96,189**
2004	**−454**	**−11,969**
2005	**25,793**	**5,786**
2006	**103,072**	**78,633**
2007	**−490**	**−14,574**
2008	**4,022**	**−13,043**
2010	**42,714**	**−4,102**
***Additional cost of QIV = USD$2***		
2001	2,349,345	4,912,836
2002	NE*	NE*
2003	1,656,956	210,065
2004	**10,766**	**−6,252**
2005	**63,259**	**29,258**
2006	217,818	174,952
2007	**10,694**	**−11,461**
2008	**19,718**	**−8,400**
2010	**97,103**	**9,483**
***Additional cost of QIV = USD$5***		
2001	5,890,874	12,308,620
2002	NE*	NE*
2003	4,159,902	551,693
2004	**44,426**	**10,900**
2005	175,659	**99,676**
2006	562,056	463,911
2007	**44,245**	**−2,124**
2008	**66,806**	**5,531**
2010	260,268	**50,238**
***Additional cost of QIV = USD$10***		
2001	11,793,422	24,634,926
2002	NE*	NE*
2003	8,331,478	1,121,073
2004	**100,527**	**39,487**
2005	362,993	217,039
2006	1,135,786	945,509
2007	**100,164**	**13,440**
2008	145,286	**28,749**
2010	532,209	118,163

# ICER = incremental cost per QALY saved by QIV. Using the threshold of 3-time gross domestic product per capita in Hong Kong as the willingness-to-pay per QALY, QIV was cost-effective with ICER USD109,671 or less (bold). A negative value of ICER indicated that QIV was less costly than TIV and gained higher QALYs.

*NE = Not effective. ICER was not calculated in 2002 because the expected reduction in infection and expected QALY gained were zero.

## Discussion

The present analysis quantified the expected reduction in TIV-unmatched influenza B infection rate, direct medical cost and indirect cost of productivity loss, and QALYs loss if QIV was available in Hong Kong during 2001–2010. The symptomatic infection rates were reduced by 191.3 (95%CI 45.1-337.5) per 100,000 elderly aged ≥65 years in the 2001–2010, comparable to the estimated reduction in the US reported by Reed et al. (ranged from 1-440 per 100,000 in 2001–2008) [2].

As demonstrated by the annual cost savings and QALYs loss averted by QIV (Table 2), and QIV would be more cost-effective than TIV if the unit cost of QIV is the same as TIV (additional unit cost = USD0) in all 9 years (except year 2002). The number of years in which QIV was more cost-effective than TIV decreased in both age groups 65–79 years and ≥80 year when the unit cost of QIV increased by USD1-10 (Table 3). Our results were different from the findings of an economic study of QIV in the US [3]. Lee et al. reported positive cost savings (including both direct and indirect costs) by QIV when the unit cost of QIV was USD5-30 more than TIV, based upon infection rate reduction reported by Reed et al. In the present analysis, QIV would cost more to gain QALYs (as indicated by a positive value of ICER) in most of the years when QIV cost more than TIV by USD1-10. The difference was mainly caused by lower healthcare costs in Hong Kong comparing to the US for inpatient (USD1,800-6,600 versus USD4,221-11,372) and outpatient care (USD49 versus USD76-102). For every case of infection averted, less direct cost of treatment was saved in Hong Kong versus US because of the lower cost of care.

In the present study, QIV was cost-effective in year 2007 at four cost levels (+USD1, +USD2, +USD5 and + USD10). The cost savings and QALYs gained by QIV were the highest in this year. It could be explained by the fact that a high percentage of circulating influenza B lineages in Hong Kong were not covered by TIV (92.9%) and the hospitalization rate for influenza B in the elderly was the highest in 2007 [1]. The cost-effectiveness of the influenza vaccine tends to be enhanced when the expected hospitalization rate without the vaccine is high [28]. The expected QALYs gained and cost savings of QIV were sufficient to overcome the economic impact of additional cost of QIV and it therefore remained cost-effective over a broad range of additional vaccine cost.

Our study was limited by using a single source (one teaching hospital) of influenza B hospital admission rates and circulating virus lineages for the calculation of population-based influenza B infection rate in Hong Kong elderly. The infection rate was estimated by the case-fatality ratio, yet this information was not available from Hong Kong. A case-fatality ratio derived from US was used, as the estimated influenza-related mortality rates were generally similar in Hong Kong and the US [29],[30]. We assumed that patients in the outpatient setting would seek care at medical clinic for one time, as Chinese older people in Hong Kong tend to perceive influenza as a serious illness [31]. Some patients would use home remedies or over-the-counter preparation to alleviate influenza symptoms, and the cost-saving of QIV might be overestimated. On the other hand, the QALYs loss of caregivers was not included in the analysis and the QALY saved by QIV might therefore be underestimated. The current cost and QALYs were calculated based upon the vaccine coverage of 39.1% and herd immunity effect was not included in the analysis. The study findings are likely to change if the vaccine coverage is improved in elderly (and therefore generates herd immunity), probably showing more prominent saving in cost and QALYs with QIV comparing to TIV. Sensitivity analysis was not conducted in the present study. Expected cost and clinical outcomes were calculated for 9 seasons using year-specific key parameters. The change of cost and clinical outcomes was found to be sensitive to the variation of key year-specific parameters (namely the percentage of circulating TIV-unmatched influenza B lineage and hospitalization rate). Using year-specific inputs has therefore served the purpose of sensitivity analysis.

## Conclusions

In conclusion, QIV could reduce direct and indirect costs of influenza B infection and gain higher QALYs when compared to TIV in the elderly population of Hong Kong. The acceptance of QIV to be cost-effective in Hong Kong elderly was highly subject to the unit cost of QIV versus TIV and the percentage of circulating TIV-unmatched influenza B lineages.
